# Genomics in Bacterial Taxonomy: Impact on the Genus *Pseudomonas*

**DOI:** 10.3390/genes11020139

**Published:** 2020-01-29

**Authors:** Jorge Lalucat, Magdalena Mulet, Margarita Gomila, Elena García-Valdés

**Affiliations:** 1 Microbiologia, Departament Biologia, Universitat de les Illes Balears, 07122 Palma de Mallorca, Spain; mmagdalena.mulet@uib.es (M.M.); marga.gomila@uib.es (M.G.); elena.garciavaldes@uib.es (E.G.-V.); 2 Institut Mediterrani d’Estudis Avançats, IMEDEA (CSIC-UIB), 07122 Palma de Mallorca, Spain

**Keywords:** *Pseudomonas*, genomics, phylogenomics, taxonomy

## Abstract

The introduction of genomics is profoundly changing current bacterial taxonomy. Phylogenomics provides accurate methods for delineating species and allows us to infer the phylogeny of higher taxonomic ranks as well as those at the subspecies level. We present as a model the currently accepted taxonomy of the genus *Pseudomonas* and how it can be modified when new taxonomic methodologies are applied. A phylogeny of the species in the genus deduced from analyses of gene sequences or by whole genome comparison with different algorithms allows three main conclusions: (i) several named species are synonymous and have to be reorganized in a single genomic species; (ii) many strains assigned to known species have to be proposed as new genomic species within the genus; and (iii) the main phylogenetic groups defined by 4-, 100- and 120-gene multilocus sequence analyses are concordant with the groupings in the whole genome analyses. Moreover, the boundaries of the genus *Pseudomonas* are also discussed based on phylogenomic analyses in relation to other genera in the family *Pseudomonadaceae*. The new technologies will result in a substantial increase in the number of species and probably split the current genus into several genera or subgenera, although these classifications have to be supported by a polyphasic taxonomic approach.

## 1. Introduction

Genomics is profoundly changing the way in which bacterial taxonomy is developing. The knowledge of the whole genome sequence of bacterial strains and analyses of their gene sequences has gained superior value over the phenotypic traits for bacterial classification and identification. Whole genome comparisons allow a more robust taxonomic framework known as taxogenomics. Digital whole genome comparisons by using average nucleotide identities (ANIs) or genome-to-genome-distance calculations (GGDCs) are the new gold standards for species circumscription, substituting experimental DNA-DNA hybridization procedures [1,2,3]. Digital DNA-DNA hybridizations (dDDHs) have many advantages over experimental data because they provide a repository of data that can be accumulative and accessible on publicly available databases. Additionally, the phylogenetic relationships among bacteria can be inferred from the nucleotide sequences of selected genes and allow a natural classification of bacteria based on their evolutionary relationships. The 16S rDNA gene sequence was initially selected in phylogenetic studies, but it is now recognized that it lacks sufficient discriminatory power to differentiate species in many genera (e.g., *Aeromonas*, *Bacillus*, *Pseudomonas*, *Streptococcus*, etc.) [4]. Therefore, other housekeeping genes have also been proposed as alternative genes for phylogenetic studies [5]. These genes have to be universal or at least present in all species in the group to be studied and can be analyzed individually, or their sequences can be concatenated and analyzed from a phylogenetic perspective. In so-called multilocus sequence analysis (MLSA), a set of seven genes was recommended [6], although it has been demonstrated that 3 or 4 gene sequences can be sufficient for the analyses in some genera, such as *Pseudomonas* [5]. MLSA is a modification of the multilocus sequence typing procedure (MLST) proposed by Maiden and collaborators [7]. More recently, with the advent of the whole genome sequence of bacterial strains, phylogenomics has contributed substantially to a modern bacterial taxonomy. Phylogenomics focuses on the study of evolutionary relationships among bacteria by multiple alignment of homologous sequences in the core genome of the bacterial group to be studied and by the inference of the corresponding phylogenetic tree. Recently, Parks and collaborators [8] proposed a standardized bacterial taxonomy (GTDB taxonomy) based on genome phylogeny by analyzing the amino acid sequences of 120 proteins encoded by 120 universal genes. In this proposal, the authors also included genomes assembled from metagenomes (MAGs) that substantially increase the diversity of bacterial species so far cultivated. MAGs might be predictive of existing bacterial species within complex biological mixtures without the need for their cultivation.

*Pseudomonas* is a diverse and complex bacterial genus that occupies many niches and environmental habitats. It is the genus of Gram-negative bacteria with the highest number of recognized species. More than 220 species have been characterized, and their taxonomic names have been validated as of the writing of this manuscript (List of Prokaryotic Names with Standing in the Nomenclature [9]). Approximately 10 new species in the genus have been described yearly in the last 10 years. It is a model organism, in which taxonomic tools have been developed and tested successfully [10]. The genus comprises three main lineages based on the 16S rRNA gene sequences that are represented by the species *Pseudomonas aeruginosa*, *Pseudomonas fluorescens* and *Pseudomonas pertucinogena*. Furthermore, 14 groups of species were initially delineated based on analyses of 3- or 4-gene nucleotide sequences in a MLSA [5,11,12]. The selected genes were: 16S rDNA, *gyrB* (gyrase B subunit), *rpoB* (B subunit of RNA polymerase) and *rpoD* (D subunit of RNA polymerase). Several of these 14 groups have also been analyzed deeper. The important group of phytopathogens represented by *Pseudomonas syringae* has been analyzed under a phylogenomic point of view by Gomila and collaborators, considering 149 genes in the core genome of 139 strains in the group, including 15 type strains [13]. Similarly, whole genome sequences have been used to clarify the taxonomy of species in the fluorescens phylogenetic group of species [14], in a comparison of *P*. *aeruginosa* and *Azotobacter* [15] and in the *Pseudomonas putida* group of species [16].

To apply a phylogenomic analysis in bacterial taxonomy, the whole genome sequences of the species type strains are needed because they are the species representatives. This goal has been almost completely achieved for the genus *Pseudomonas* in various publications, which have been summarized recently by Hesse and collaborators [17] in the frame of the GEBA (Genome Encyclopedia of Bacteria and Archaea) project initiated by the Joint Genome Institute [18]. A total of 166 species or subspecies type strains were analyzed by comparing 100 amino acid sequences of proteins encoded by housekeeping monocopy orthologous genes of the core genome of the genus. In the aforementioned Genome Taxonomy Database, Parks and collaborators [8] included 175 *Pseudomonas* species type strains, together with 4971 non-type strains and genomes retrieved from several metagenomics assemblies (MAGs).

The currently accepted taxonomy of 217 species type strains in the genus *Pseudomonas* based on a 4-gene MLSA study is presented in this study, whose main aim is the comparison of the current taxonomy with the results obtained by genomic analyses in previous studies performed by our research group [11,16] and those obtained by Hesse et al. [17] and Parks et al. [8]. The results demonstrate that: (i) several named species are synonymous and have to be reorganized in a single genomic species; (ii) many strains assigned to known species have to be proposed as new genomic species within the genus; and (iii) the main phylogenetic groups defined by the 4-gene MLSA analysis are concordant with the groupings in the genomic analyses. The boundaries of the genus *Pseudomonas* are also discussed based on phylogenomic analyses in relation to other genera in the family *Pseudomonadaceae*. Furthermore, the possibility of applying the genomic subspecies boundaries proposed by Meier-Kolhoff and collaborators [19] is presented by using *Pseudomonas chlororaphis* genomes as a case study.

## 2. Materials and Methods

The list of species type strains studied and their genome accession numbers in the National Center for Biotechnology Information (NCBI) or the Joint Genome Institute (JGI) websites are given in Supplementary Table S1. It includes 217 species type strains in the genus *Pseudomonas*, together with strains of the sister genera *Azotobacter*, *Azomonas*, *Enteromonas*, *Oblitimonas*, *Thiopseudomonas* and *Ventosimonas*. *Cellvibrio japonicus* and *Escherichia coli*-type strains were included as outgroups.

The 16S rDNA sequence accession numbers in the NCBI database are indicated in Table S1 and have been analyzed as previously described by Mulet and collaborators [5]. MLSA of 4 housekeeping genes were performed by the concatenation of their respective partial gene sequences as previously described [5,11]: 16S rDNA, *gyrB* (gyrase B subunit), *rpoB* (B subunit of RNA polymerase) and *rpoD* (D subunit of RNA polymerase). Gene sequences were retrieved from the NCBI database or were extracted from the respective genomes. If they were not available in public databases, the sequences were obtained in the present study following procedures previously described [20]. Phylogenetic distances were calculated by the Jukes–Cantor algorithm, and trees were constructed by neighbor-joining using Molecular Evolutionary Genetics Analysis (MEGA5) software [21]. Bootstrap values were calculated in percentage from 1000 replications. The stablished species cutoff is 97% [5]. The maximum likelihood phylogenetic tree of the concatenated analysis was also constructed using the PhyML 3.0 software [22]. PartitionFinder 2 was previously used to estimate the best evolutionary model, the general time reversible model (GTR + I + R), using the Bayesian information criterion [23]. Phylogenetic tree obtained was also visualized by MEGA7 software using the midpoint rooting approach.

Average nucleotide identities based on BLAST (ANIbs) were calculated at the JSpecies website (http://jspecies.ribohost.com/jspeciesws/) [24,25] for species delineation or to assess the synonymy between two type strains detected by the MLSA and phylogenomic analyses. Two strains were considered members of the same genomic species when their ANIb value was equal to or higher than 95%.

Phylogenomic analyses for species delineation are discussed by the following 3 methodologies. (i) Hesse and collaborators [17] selected 100 monocopy protein sequences common to all *Pseudomonas* strains as phylogenetic markers with the shortest Robinson–Foulds distance. These protein families are considered to be the least affected by horizontal gene transfer. Most of these housekeeping proteins are ribosomal proteins. Phylogenies were inferred by maximum likelihood. (ii) Data for the phylogenies inferred from the concatenation of the amino acid sequences of 120 ubiquitous monocopy proteins were analyzed in the Genome Taxonomy Database (GTDB) website in September 2019 (http://gtdb.ecogenomic.org/) [8,26]. The 120 selected proteins are mainly ribosomal proteins, and only 40 were coincident with those used by Hesse and collaborators [17] in their phylogenetic analyses. The phylogenetic distances were calculated by the relative evolutionary divergence (RED) after normalization for lineage-specific rates of evolution. In addition to the type strains, 2416 genome sequences of the Genome Taxonomy Database (GTDB) of cultivated strains and MAGs assigned to species in the genus *Pseudomonas* as of September 2019 were also considered in the taxonomic analysis. (iii) In the *Pseudomonas chlororaphis* case study, we also followed the procedures described by Meier–Kolthoff and Göker [27] based on the genome BLAST distance phylogeny method (GBDP), as implemented in the TYGS (Type Strain Genome Server) platform (https://tygs.dsmz.de/) [28].

Genus boundaries were assessed by the percentage of conserved proteins index (POCP) [29] with the assumption that two species of the same genus should share at least half of their proteins. The percentage index was calculated as $\left[\frac{C1+C2}{T1+T2}\right]*100$, where C1 and C2 are the conserved number of proteins in the two genomes compared, and T1 and T2 are the total number of proteins in the two genomes. The genus cutoff index established was 50%. The number of proteins shared by genome sequences available at the JGI website was calculated with the “Phylogenetic Profiler for Single Genes” tool (https://img.jgi.doe.gov/) by setting similarity cutoffs with a maximal E-value of 1e-5 and minimal percent identity of 50% [30]. Details of genus boundaries in the GTDB taxonomy are given in the Parks and collaborators study [8].

## 3. Results

### 3.1. 16S rDNA Phylogeny

The 16S rDNA sequence is mandatory for the description of a new species, and its analyses constitute the backbone of the actual bacterial taxonomy. As a universal marker, it permits the ascription of a strain to the genus and allows comparisons between very divergent bacteria. The lowest similarity among the 220 *Pseudomonas* species type strains studied was 91.2%. Excluding members of the *P. pertucinogena* group, the lowest similarity was 92.2% between *Pseudomonas thermotolerans* and *Pseudomonas duriflava*. The 16S rDNA sequence allowed differentiation from the sister genera *Cellvibrio*, *Oblitimonas*, *Thiopseudomonas* and *Ventosimonas*. The three main *Pseudomonas* lineages (*P. aeruginosa*, *P. fluorescens* and *P. pertucinogena*) are also separated and supported with relatively high bootstrap values (Supplementary Figure S1). The genus *Azotobacter* is embedded in the *Pseudomonas* genus, but in the borderline, with similarities ranging from 93.6% to 97.2% among strains in the aeruginosa and fluorescens lineages, as shown in the Supplementary Materials (Table S2). The low differentiation value of these sequences for species differentiation can be exemplified, for instance, for *Pseudomonas lurida*. The type strain shares more than 99% identity in its 16S rDNA sequence with 40 other species type strains in the *P. fluorescens* group of species, constituted by 74 species and 3 subspecies. The situation is more complex when other non-type strains are included in the analyses and, therefore, other gene sequences were then used for species differentiation. In general, it is accepted that identities lower than 98.6% between 2 strains imply that the strains belong to different species, but a species cutoff cannot be established at higher levels of identity, such as those found in the *P. fluorescens* group of species previously discussed. A clear gap could not be detected in the similarity values for the species differentiation (Supplementary Figure S2) due to the high sequence conservation of the 16S rDNA. As depicted in Supplementary Figure S1, the bootstrap values supporting the recognized phylogenetic groups or subgroups within the genus are very low (lower than 10 in many bifurcating branches), but they allow differentiation.

### 3.2. Four-Gene MLSA

**Figure 1:**
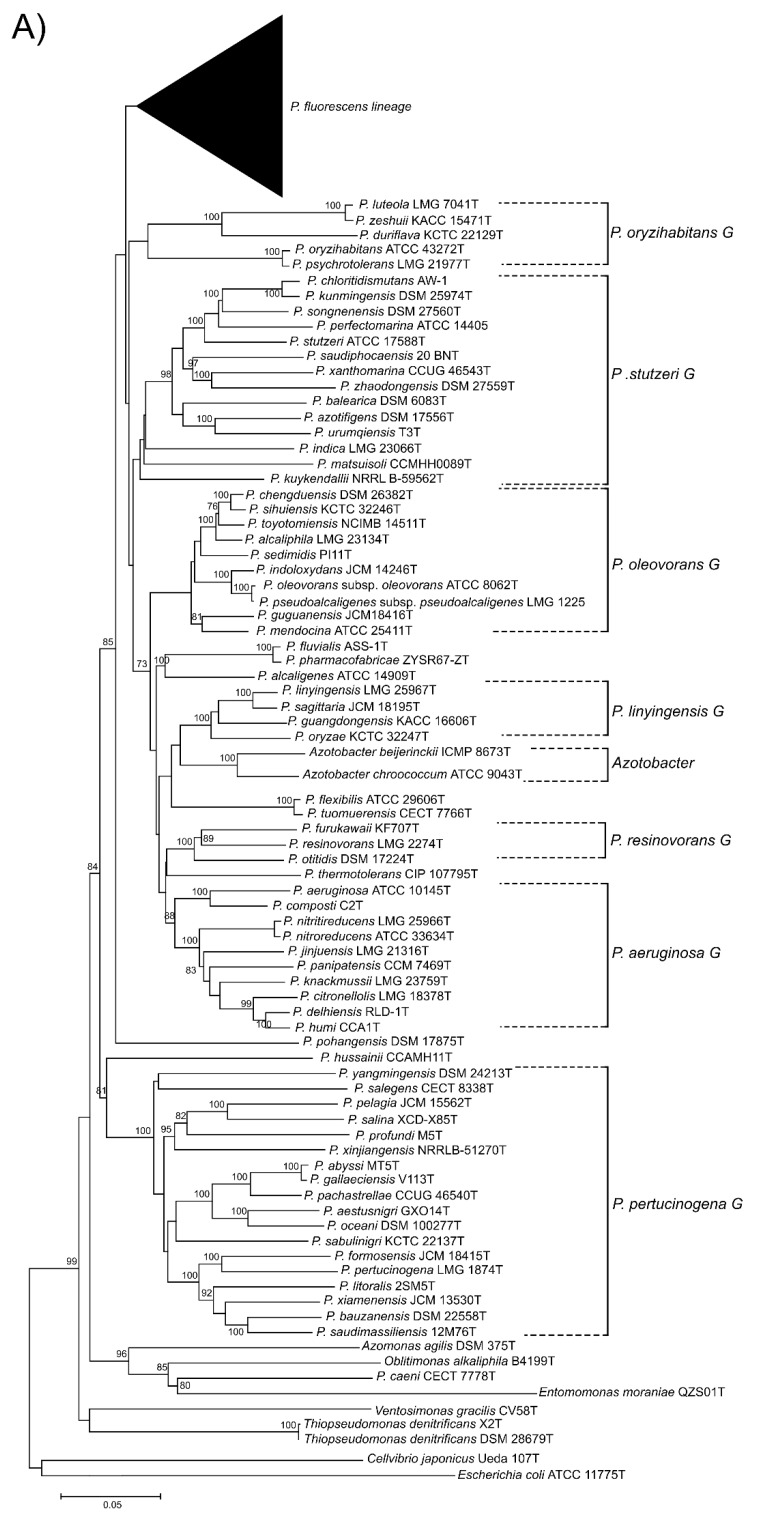

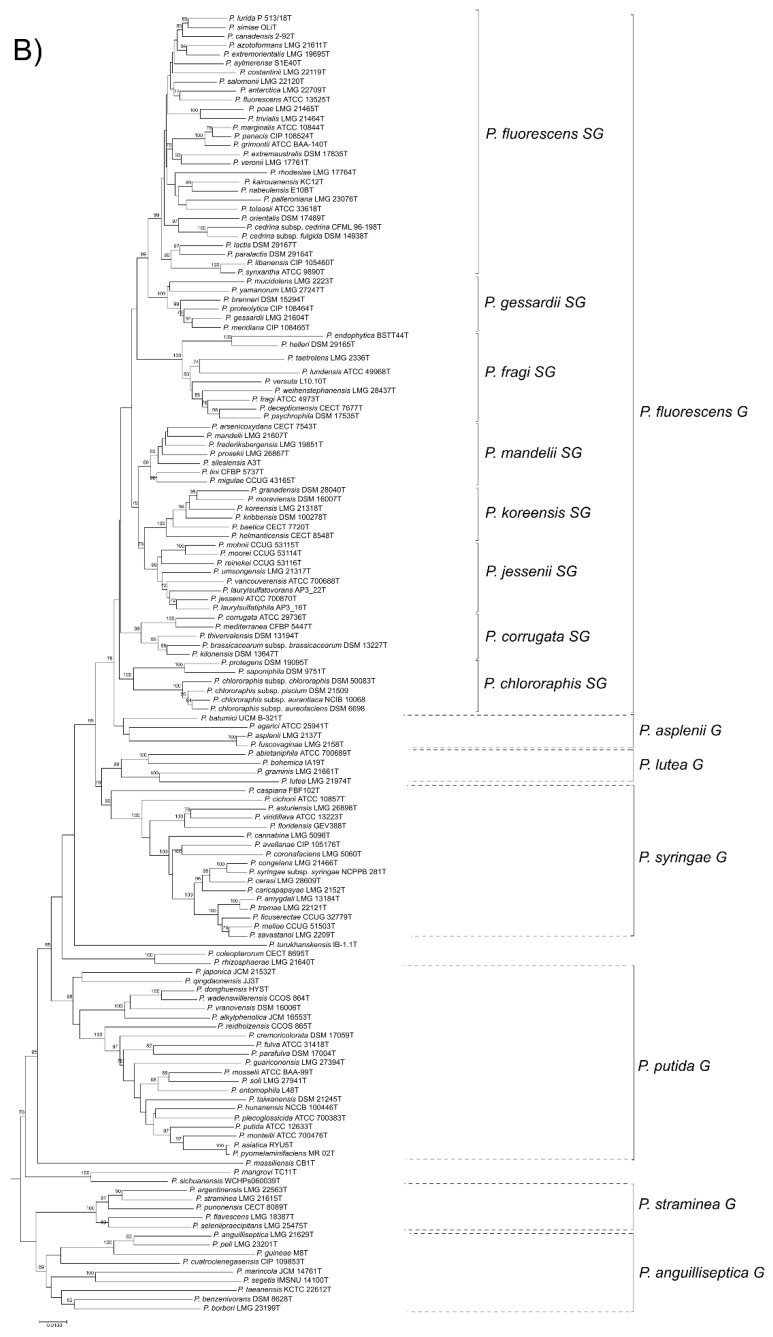
Phylogenetic tree based on the 4-genes MLSA for the 227 species or subspecies type strains analyzed using neighbor-joining reconstruction with Jukes–Cantor distances. (**A**) *P. aeruginosa* and *P. pertucinogena* lineages. (**B**) *P. fluorescens* lineage. Bootstrap values higher than 70% are indicated on the nodes. Bars indicate sequence divergence.

**Figure 2:**
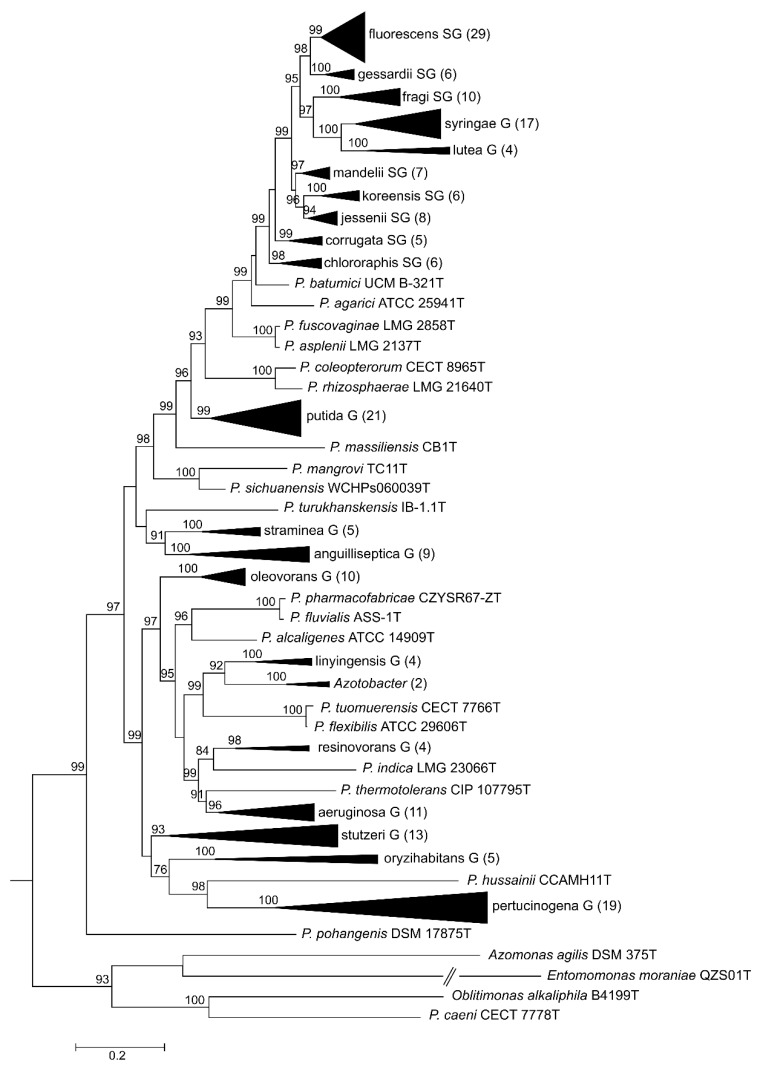
Maximum likelihood phylogenetic tree constructed with PhyML 3.0 based on the 4-genes MLSA for the different groups and subgroups defined in the *Pseudomonas* genus and closest-related genera. GTI+I+R was selected as the best evolutionary method. Number of species in each collapsed branch are indicated in brackets. Bootstrap values higher than 70% are indicated on the nodes. Bar indicates sequence divergence.

Here, we present a complete MLSA based on the concatenated partial gene sequences of the four selected genes (16S rDNA, *gyrB*, *rpoB* and *rpoD*) for the 216 *Pseudomonas* species and subspecies type strains under study (Figure 1 and Figure 2 and Supplementary Figure S3). Figure 1 shows the tree based on the Jukes–Cantor index and constructed by neighbor-joining. Figure 2 and Supplementary Figure S3 shows the maximal likelihood phylogenetic tree using PhylML based on the best evolution model obtained, the GTR+I+R index. The sister genera are clearly separated from the *Pseudomonas* branches, but species of *Azotobacter* form a distinct branch within *Pseudomonas*. As previously demonstrated [5,12], the genus can be divided into three main lineages (*P. aeruginosa*, *P. fluorescens* and *P. pertucinogena*). As indicated in Figure 1, the *P. fluorescens* lineage comprises 5 phylogenetic groups (*P.* *fluorescens*, *P. asplenii*, *P. lutea*, *P. syringae* and *P. putida*,); the *P. aeruginosa* lineage comprises 8 phylogenetic *Pseudomonas* groups (*P. straminea*, *P. anguilliseptica, P. oryzihabitans*, *P. stutzeri*, *P. oleovorans*, *P. resinovorans*, *P. aeruginosa* and *P. linyingensis*) and the genus *Azotobacter*. Eleven species are scattered along the tree (*P. coleopterorum*, *P. rhizospherae*, *P. massiliensis*, *P. mangrovi*, *P. sichuensis*, *P. fluvialis*, *P. pharmacophabricae*, *P. alcaligenes*, *P. thermotolerans*, *P. pohangensis* and *P. caeni*). The groupings of species are identical in both trees and only few differences can be observed in the branching order. The main difference is the inclusion of the lutea and syringae groups within the fluorescens group in Figure 2.

The fluorescens group contains 72 species and 3 subspecies. It is further subdivided into 8 phylogenetic subgroups (Figure 1). Almost all species type strains are differentiated at a species cutoff of 97% in the 4-gene MLSA, as demonstrated in Supplementary Table S3 and Figure S2. In only 13 comparisons, type strains shared a sequence similarity higher than 97% and were suspected to be strains of the same species (Table 1). To confirm this result, the genomes were compared by the ANIb and GGDC methods. In all cases, the ANI was higher than 95% (the species cutoff in this study); 94–96% ANI is the threshold suggested by Richter and Rosselló-Móra [3]. The GGDC was higher than 70% (the species cutoff), confirming that the pairs of type strains should be considered synonymous, as discussed later.

### 3.3. Phylogeny Based on 100 Gene Sequences

Hesse and collaborators [17] included 163 species and 3 additional subspecies type strains from the 180 species recognized at the moment of performing the analyses. A maximum likelihood phylogeny of the type strains was based on analyses of 100 orthologous single-copy proteins (100-gene MLSA). Thirteen phylogenetic groups of type strains were described. As indicated in Supplementary Table S4, the grouping of strains was highly concordant with those devised by the 4-gene MLSA, with few exceptions [17]. In agreement with the 4-gene MLSA, at least 7 pairs of species type strains should be considered synonymous, as well as a group of four species in *the P. syringae* group (*P. ficuserectae*, *P. meliae*, *P. savastanoi* and *P. amygdali*), as already proposed in a previous study [13]. The possible species status of the groups determined by 100-gene MLSA was confirmed by ANIb analyses of their genomes. Analyses of a total of 1224 genomes, including non-type strains, also demonstrated 394 potential new species that were assigned to individual ANI clusters (“cliques”) in the JGI website by their gANI and genome alignment fraction (AF) calculations [17].

### 3.4. Analyses Performed at the Genome Taxonomy Database (GTDB Taxonomy)

**Figure 3:**
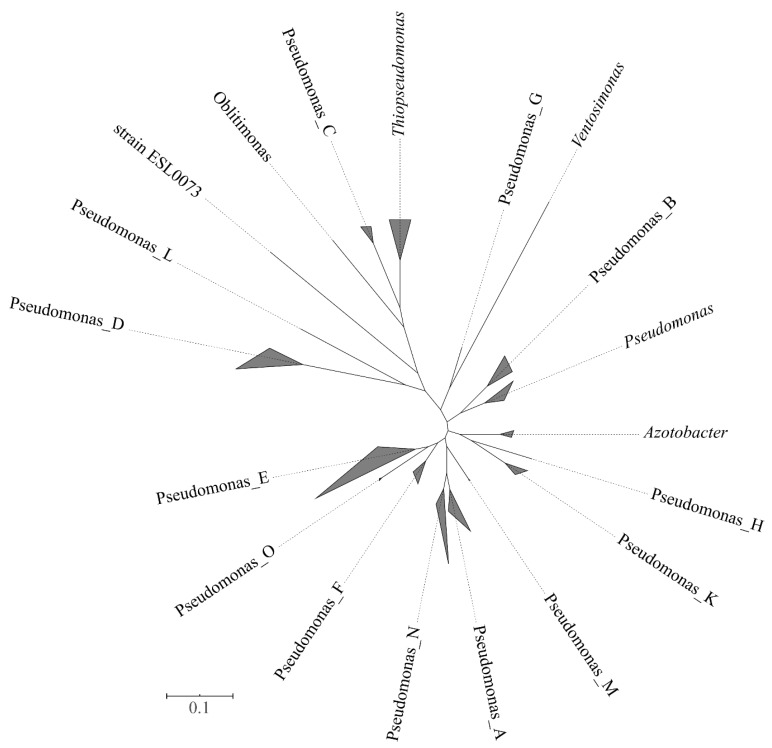
Phylogenetic tree of the *Pseudomonadaceae* genera based on the GTDB taxonomy. Triangles are proportional to the sequence divergence among species included in each genus. Bar indicates sequence divergence.

Five thousand one hundred seventy-one genomes were included in the family *Pseudomonadaceae*. At the time of performing the analyses (September 2019), 5146 *Pseudomonas* genomes were available. They included most of the type strains (148) whose genomes were so far sequenced, together with non-type strains and genomes retrieved from metagenomes (MAGs). For the phylogenetic analyses, a set of 2416 genomes was selected. For instance, from the 2744 genomes of *P. aeruginosa*, only 14 were included in the analyses by the Annotree on the website. The methods implemented in the GTDB website allowed the differentiation of 351 *Pseudomonas* clusters at the species level. The normalized relative evolutionary divergence method indicated that the family *Pseudomonadaceae* included the sister genera *Azotobacter*, *Oblitimonas*, *Thiopseudomonas* and *Ventosimonas* that were embedded within *Pseudomonas* species in the phylogenetic tree (Figure 3). A total of 19 phylogenetic clusters at the genus level can be delineated, 15 of them constituted exclusively by *Pseudomonas* strains. The group including the type species of the genus, *P. aeruginosa*, and retained the genus name *Pseudomonas*. Each of the 14 potential new genera was labeled in the GTDB website by a letter, without giving them a formal taxonomic rank (e.g., Pseudomonas_A, Pseudomonas_B, through Pseudomonas_N). Some of the proposed genera were represented by a single strain. For instance, Pseudomonas_C was represented by the *Pseudomonas caeni* type strain, and Pseudomonas_N was represented by the *Pseudomonas indica* type strain. A very good correspondence was found between the species grouped in the proposed new genera and the main phylogenetic groups previously delineated in the 4- and 100-gene MLSAs, with few exceptions (Supplementary Table S4). The number of known and putative new species in each group is indicated in Table 2. Pseudomonas_E was the branch with the highest number of species (404), which corresponded to the fluorescens lineage (anguilliseptica, fluorescens, lutea, putida, straminea and syringae groups) and oleovorans group in the 4-gene MLSA and in the study performed by Hesse and collaborators [17]. Pseudomonas_E ranked in second place in the number of species in the GTDB taxonomy, following the genus *Streptomyces* with 470 species.

### 3.5. Analysis of the Percentage of Conserved Proteins (POCP)

We calculated the POCP index for representative strains of 7 of the main phylogenetic groups detected by MLSA and with representatives of the sister genera. The indexes obtained between *P. aeruginosa* DSM 50071^T^ and the representative type strains were in many instances in the borderline for the proposed genus cutoff of 50%: 60% with *P. fluorescens*; 57% with *P. alcaligenes*; 53% with *P. stutzeri*; 50% with *P. putida*; 50% with *P. oryzihabitans*; 48% with *P. syringae*; 45% with *Azotobacter vinelandii* DJ; 36% with *Oblitimonas*; and 21% with *Cellvibrio*. *A. vinelandii* DJ was separated from the *Pseudomonas* species studied, with indexes in the range 38–45%, which is below the 50% cutoff, suggesting that *Azotobacter* is a clearly distinct genus. The outgroup was represented by *Cellvibrio japonicus*, with indexes of 20–24% with *Pseudomonas* spp.

### 3.6. Pseudomonas Chlororaphis Case Study

**Figure 4:**
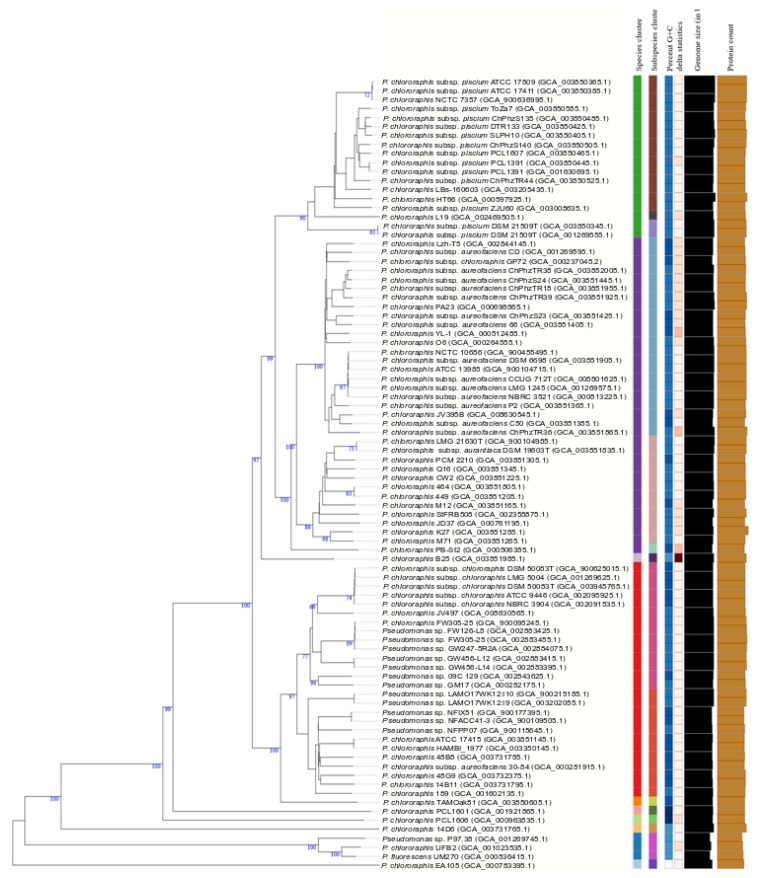
*P. chlororaphis* species and subspecies delineation based on the GBDP phylogenetic analyses retrieved from the TYGS website. The tree was inferred with FastME 2.1.6.1 [32] from GBDP distances calculated from genome sequences. The branch lengths are scaled in terms of GBDP distance formula d5. The numbers above branches are GBDP pseudo-bootstrap support values >60% from 100 replications, with an average branch support of 83.0%. The tree was rooted at the midpoint [33].

Strains in the *P. chlororaphis* subgroup formed a clear phylogenetic branch in the fluorescens group in the 4-gene MLSA, in the study of Hesse et al. [17] and in the GTDB analyses. The subgroup includes the species *Pseudomonas protegens*, *Pseudomonas saponiphila* and *P. chlororaphis*. *P. chlororaphis* is divided into 4 subspecies in the current taxonomy (*aurantiaca*, *aureofaciens*, *chlororaphis* and *piscium*) [12] and the group was selected as a case study to test the recently described methodology proposed by Meier–Kolthoff and collaborators [19,27] for bacterial species and subspecies delineation (TYGS). Eighty-eight complete or draft genomes of strains previously identified as *P. chlororaphis* or detected as closely related genomes in this study were retrieved from the NCBI or the JGI databases (see accession numbers in Figure 4 and Supplementary Figure S4). Ten species clusters were delineated as shown in Figure 4, with a species threshold of 70%. Eighteen strains in *P. chlororaphis* subsp. *piscium* were considered a separate species joining the rest of the *P.* chlororaphis strains at a level lower than 60% in their dDDH. This group contained 3 putative subspecies at the stablished cutoff of 80%. Strains of the subspecies *aureofaciens* and *aurantiaca* were separated in a different species and were considered subspecies in a new species. Strains of *P. chlororaphis* subsp. *chlororaphis* formed a single branch at a cutoff of 70% dDDH. The species delineation was concordant with the 4-gene MLSA at a threshold of 97% and with the ANI analyses at a threshold of 95% (Supplementary Figure S4). However, the GTDB tool did not distinguish *P. chlororaphis* subsp. *aureofaciens*, nor subsp. *aurantiaca*, as a separate subspecies within *P. chlororaphis* and included the type strains of both subspecies in the species “Pseudomonas_E piscium”.

Eight strains were not identified as *P. chlororaphis* and were considered members of 6 potential new species: (1) strain EA105 in the closely related koreensis subgroup, (2) strains P97.38, UFB2 and UM270 in the corrugata subgroup, and (3) strain 14D6, strain PCL1601, strain B25 and strain PCL1606 in the chlororaphis subgroup were considered 4 potential new species. The GGDC value was lower than 70%, and the 4-gene MLSA was lower than 95% with any species type strain, confirming the TYGS identification, and all were located in the 3 trees in the same phylogenetic branch. Strains P97.38, UFB2 and UM270 showed GGDC values of 80.6% to 92.9% and a 4-gene MLSA value of 98.4–99.4%, and the GTDB taxonomy identified them as a new species, Pseudomonas_E chlororaphis_E, demonstrating that they formed a homogeneous genomic group at the species level. The ANIb value for these 3 strains was 97.39–98.97%. Strain EA105 was identified in the GTDB taxonomy as Pseudomonas_E chlororaphis_A and strain PCL1601 as Pseudomonas_E chlororaphis_D, confirming the good correlation among the four methods.

Twelve genome sequences deposited as *Pseudomonas* sp. were classified as *P. chlororaphis* subsp. *chlororaphis*. Twenty strains deposited as *P. chlororaphis* can now be assigned genomically to *piscium*, *aureofaciens*, *aurantiaca* or *chlororaphis* subspecies. Only 2 strains of 45 deposited with identification at the subspecies level were located in different subspecies in the TYGS tree. In summary, 74 genomes deposited as *P. chlororaphis*, 13 as *Pseudomonas* species and 1 as *P. fluorescens* were phylogenetically divided into 10 species, one with 2 subspecies and 2 more different species, with 3 subspecies each.

## 4. Discussion

### 4.1. Species and Subspecies Delineation

The currently available tools for species genomic delineation were not available at the time of proposing new species in the last century and have led to confusion in the description of several species. The need for DDH experiments has been traditionally inferred after the previous comparison of the 16S rDNA sequences to select the strains to be hybridized, and the low differentiation power of the 16S ribosomal gene sequence has led in some cases to inconsistencies. For instance, when *P. psychrophila* was described in 2002 [34], the 16S rDNA sequence of *P. oryzihabitans* described in 1985 was not included in the analysis; therefore, the experimental DNA–DNA hybridization between the *P. psychrophila* and *P. oryzihabitans* type strains was not performed. The results presented in our study demonstrate that both species are the same genomospecies and have to be considered synonymous; consequently, *P. psychrophila* is a later heterotypic synonym of *P. oryzihabitans*. The poor differentiation power of the 16S rDNA sequence led to the proposal of other genes for phylogenetic studies of close-related species. The MLST scheme initially proposed by Maiden [7] for bacterial typing was transformed to a MLSA by other authors. The protein-coding genes selected for the phylogenetic analyses are housekeeping genes considered not prone to horizontal gene transfer. In the analysis performed by Hesse and collaborators, the Robinson–Fould distances were calculated for protein families in the Pseudomonas spp. core genome. The genes selected belonged to the group of genes least affected by horizontal gene transfer [17]. Those genes routinely used in *Pseudomonas* taxonomy are related to replication and translation. The *rpoD* and *gyrB* genes were used for the first time by Yamamoto and collaborators [35] and the *rpoB* gene was later proposed by Tayeb and collaborators [36]. They have been used widely in the descriptions of new species within the genus.

Another example is our case study, the taxonomy of *P. chlororaphis.* The species has suffered several changes after its inclusion in the Approved Lists of Bacterial Names [37], in which three closely related species were recognized: *P. chlororaphis*, *P. aurantica* and *P. aureofaciens*. In 1989, Johnson and Palleroni proposed that *P. aureofaciens* should be considered a later heterotypic synonym of *P. chlororaphis* in a study based on DNA–DNA hybridizations and phenotypic traits [38]. In the Second Edition of Bergey’s Manual of Systematic Bacteriology [10], Palleroni proposed that *P. aureofaciens* and *P. chlororaphis* strains should be considered subspecies in the same species, *P. chlororaphis*. Later, in a polyphasic analysis performed in 2007 by Peix and collaborators [39], the authors concluded that *P. aurantiaca* should be considered a third subspecies within *P. chlororaphis*. In 2010, Burr et al. proposed a fourth subspecies, *P. chlororaphis* subsp. *piscium* [40], to accommodate two strains isolated from the intestines of freshwater fish. The experimental DNA–DNA hybridization between the subspecies *piscium* and *aurantiaca* and *chlororaphis* was 81% and 80%, respectively, whereas the value between *P. piscium* and *P. aureofaciens* type strains was 74%. The combination of 6 phenotypic traits can be used for subspecies differentiation, but only 2 characteristics are different between *P. chlororaphis* subsp. *piscium* and *P. chlororaphis* subsp. *chlororaphis* (arginine dihydrolase reaction and 3-hydroxybenzoate assimilation). A 4-gene MLSA study placed the four subspecies type strains in the same species, with similarity values between 97.9% and 98.4%. The subsp. *chlororaphis* and *piscium* were clearly differentiated in the study of Hesse, in the GTDB website, with the TYGS procedure and by the ANIb analysis. The situation was not as clear when strains of the subsp. *aurantiaca* and subsp. *aureofaciens* were analyzed. In the study of Hesse and collaborators, they were assigned to the same species with 2 subspecies, ANI could not differentiate both subspecies, and in the GTDB website, aurantiaca and aureofaciens strains were classified as members of “Pseudomonas_E piscium”. The difficulties in the species/subspecies differentiation in the *P. chlororaphis* study confirm the utility of DNA sequence-based classification of strains, even when they are closely related, but also that other traits must be considered in bacterial systematics and that polyphasic approaches are still needed, at least in some cases. The definitive classification of strains in the *P. chlororaphis* group requires further study.

ANIb and GGDC are so far the best approaches to delineate bacterial species. However, to infer the phylogeny of the species in the genus *Pseudomonas* and sister genera, other methods should be applied. Accepting that the 16S rDNA sequence is sufficient to separate genera but not sufficiently discriminate the phylogeny of *Pseudomonas* species, we should accept that MLSA studies provide the best tool thus far. The question is what genes have to be selected and how many gene sequences are needed to establish a stable *Pseudomonas* taxonomy. The 4-gene MLSA cutoff of 97% is well correlated with ANI and GGDC and is easy to implement in laboratories. If the genome sequences are available, several alternatives exist: (i) the species identification tool, specI [41], selects 40 universal genes; (ii) the GTDB taxonomy selects 120 universal genes; iii) Hesse and collaborators selected 100 monocopy genes (only 40 also included in GTDB); and Garrido-Sanz et al. [14] and Gomila et al. [13] selected 1334 genes and 149 monocopy genes, respectively, from the core genome of the groups studied, *P. fluorescens* and *P. syringae*. It seems reasonable that in the study of the phylogeny of a single species, genes found in most individuals in the species, which we can consider the core set of genes for that species, are the genes that determine those properties characteristic of all members of the species and should be selected. In the same way, for each phylogenetic group, the core genome should provide the best approach.

### 4.2. Genus Delineation

The search for molecular tools for genus delineation has not received as much attention as the species boundaries received. Currently, a 94.5% threshold in the 16S rDNA similarity is the recommended value to determine the affiliation of a bacterial strain to an existing or a new genus [42,43]. As indicated in Supplementary Table S2, the lowest value among *Pseudomonas* type strains is 91.2%. However, excluding the type strains of the 17 species in the *P. pertucinogena* group (Pseudomonas_D in the GTDB taxonomy)*,* the similarity values among *Pseudomonas* species were higher than 95%, the recommended genus threshold. All indexes indicate that the lineage of *P. pertucinogena* should be considered a different genus at the same taxonomic rank as the sister genera in the present study.

*Azotobacter* species conform to a phenotypically well-defined genus, but phylogenomic studies demonstrate their close relationship to *Pseudomonas*. The 16S rDNA sequences are in the genus borderline, and several authors have proposed their inclusion in the same genus [44,45]. Parks and colleagues used the relative evolutionary divergence (RED) values after normalization in the GTDB taxonomy to divide *Pseudomonas* species into 15 genera that were basically coincident with the phylogenetic groups defined by the 4-gene and 100-gene phylogenies. The GTDB taxonomy considers *Azotobacter* as an independent genus but embedded in the proposed 15 *Pseudomonas* genera. The main difficulty is where to establish the genus thresholds for *Pseudomonadaceae*. Qin et al. [29] proposed the percentage of conserved proteins (POCP) as a genomic index for genus differentiation based on the assumption that two species of the same genus should share at least half of their proteins. In our analyses, *Azotobacter* is clearly a different genus by the POCP index, but in the lower borderline (38–48%). The same situation was detected in the analysis of species in the stutzeri group (47–53%). A difficulty in considering this index in the genus *Pseudomonas* might be the substantial differences in the genome sizes of the *Pseudomonas* spp. For instance, *P. aeruginosa* PA7 contains 6369 proteins, whereas *P. stutzeri* A15 contains 4200 proteins, a 33% smaller genome. The enormous diversity in the genomes of a single species can also be exemplified by *P. aeruginosa*. Its core genome is composed of 665 genes, which is only 1% of the pangenome of the species [46]. The GTDB taxonomy has an advantage over the phylogenies currently in use because the taxonomic ranks are normalized (for details see [8]).

## 5. Conclusions

In the words of Palleroni, the genus *Pseudomonas* underwent a “big bang” when 16S rRNA comparisons were introduced in bacterial taxonomy. As a result, many species were transferred to existing or new genera. Paraphrasing Palleroni’s words, taxonomy based on phylogenomics will led to a second “big bang” of the currently accepted genera giving rise to many new species and genera and/or subgenera. A thorough complete taxonomic analysis is needed to solve this situation, including the genomes of those species type strains not yet sequenced in *Pseudomonadaceae*. At least 10 type strains have to be sequenced and will no longer be considered “orphans” in the sense of this term used earlier for species without their 16S rDNA sequence. As in many other occasions, *Pseudomonas* will serve as a model organism for modern bacterial taxonomy, and will help to clarify the taxonomy of other genera. The difficulties in bacterial taxonomy can be summarized in the thoughts of Stanier, who defined taxonomy as “the art of biological classification” [47].

## Figures and Tables

**Table 1:** List of *Pseudomonas* species currently accepted in taxonomy that should be considered synonyms after a phylogenetic analysis and their reclassification. ANIb values are the averages of the bidirectional comparisons.

Synonyms	ANIb ^a^	4-gene MLSA	Reclassification
*P. asplenii*	97.91	99.6	*P. aspleniii*
*P. fuscovaginae*			
*P. meliae*	>97	98.2	*P. amygdali*
*P. amygdali*		100	
*P. savastanoi*		98.3	
*P. ficuserectae*		98.6	
*P. asiatica*	99.91	100	*P. asiatica*
*P. pyomelaninifaciens*			
*P. chloritidismutans*	96.29	99.8	*‘P. chloritidismutans’ ^b^*
*P. kunmingensis*			
*P. oleovorans* subsp. *oleovorans*	96.75	99.9	*P. oleovorans*
*P. indoloxidans*			
*P. flexibilis*	100	99.5	*P. flexibilis*
*P. tuomuerensis*			
*P. fluvialis*	98.46	99.2	*P. fluvialis*
*P. pharmacofabricae*			
*P. nitritireducens*	*-*	99.4	*P. nitroreducens*
*P. nitroreducens*			
*P. citronellolis*	95.9	99.5	*P. citronellolis*
*P. humi*			
*P. oryzihabitans*	97.70	99.7	*P. oryzihabitans*
*P. psychrotolerans*			
*P. luteola*	97.60	99.3	*P. luteola*
*P. zeshuii*			
*P. abyssi*	97.10	99.7	*P. gallaeciensis*
*P. gallaeciensis*			

^a^ Genomes were not available in public databases; ^b^ *P. chloritidismutans* was considered a member of *P. stutzeri* gv. 3 by Cladera et al., 2006. [31].

**Table 2:** Proposed genera defined by the GTDB taxonomy in the family *Pseudomonadaceae* compared with the currently recognized species, phylogenetic groups and genera based on the 4-genes MLSA. The number of species included in each group are also indicated.

Proposed Genera and Species in the GTDB Taxonomy		Accepted Taxonomy and Phylogenetic Groups	
**Genera**	**nr. species**	**Genus, group (G) or representative species**	**nr. species**
*Azotobacter*	3	*Azotobacter*	8
*Oblitimonas*	1	*O. alcaliphila*	1
*Pseudomonas*	14	aeruginosa G	10
Pseudomonas_A	38	stutzeri G	12
Pseudomonas_B	7	oryzihabitans G	5
Pseudomonas_C	3	*P. caeni*	1
Pseudomonas_D	18	pertucinogena G	18
Pseudomonas_E	404	anguiliseptica G	142
		fluorescens G	
		lutea G	
		putida G	
		oleovorans G	
		straminea G	
		syringae G	
Pseudomonas_F	9	*P. resinovorans*	4
Pseudomonas_G	1	*P. thermotolerans*	1
Pseudomonas_H	1	*P. flexibilis*	1
Pseudomonas_K	4	linyingensis G	4
Pseudomonas_L	1	*P. hussainii*	1
Pseudomonas_M	2	*P. indica*	1
Pseudomonas_N	2	*P. azotifigens*	1
Pseudomonas_O	2	*P. kuykendallii*	1
*Thiopseudomonas*	2	*T. denitrificans*	1
strain ESL0073	1	-	0
*Ventosimonas*	1	*V. gracilis*	1
**Total number of species**	**514**		**213**

## Supplementary Materials

The following are available online at https://www.mdpi.com/2073-4425/11/2/139/s1, Figure S1. Phylogenetic tree based on the 16S rDNA sequences of the species and subspecies type strains under study. (A) *P. fluorescens* lineage, (B) *P. aeruginosa* and *P. pertucinogena* lineages. Bootstrap values higher than 50% are indicated on the nodes. Figure S2. Pairwise similarities of the concatenated 4-genes partial sequences (A) and the 16S rDNA sequences (B) among the studied type strains. Figure S3. Maximum likelihood phylogenetic tree based on the 4-genes MLSA for the 227 species and subspecies type strains under study. (A) *P. aeruginosa* and *P. pertucinogena* lineages. (B) *P. fluorescens* lineage. Bootstrap values higher than 70% are indicated on the nodes. Bars indicate sequence divergence. Figure S4. UPGMA dendrogram of the ANIb similarities among the *P. chlororaphis* strains studied. Table S1. Species type strains analyzed in this study. Individual genes or genome accession numbers are also indicated. Table S2. 16S rDNA similarity matrix for all species and subspecies of *Pseudomonas* type strains studied; closest genera were also included. Table S3. Four-gene MLSA similarity matrix (16S rDNA, *gyrB*, *rpoB* and *rpoD*) for all species and subspecies of *Pseudomonas* type strains studied; closest genera were also included. Table S4. Correlation among the 4-genes, 100-genes MLSAs and the genera proposed in the GTDB taxonomy. According to the GTDB taxonomy the proposed genus Pseudomonas_E could be also subdivided in different clusters as highlighted with different colors.
