# Supplementary material for: Genomics in Bacterial Taxonomy: Impact on the Genus Pseudomonas
Source: Genes (Basel). 2020 Jan 29;11(2):139. doi: 10.3390/genes11020139 (PMC7074058; doi:10.3390/genes11020139)
Supplement: Supplementary file 1 [file genes-11-00139-s001.zip › FigureSupplementaryS1_S4.pdf]

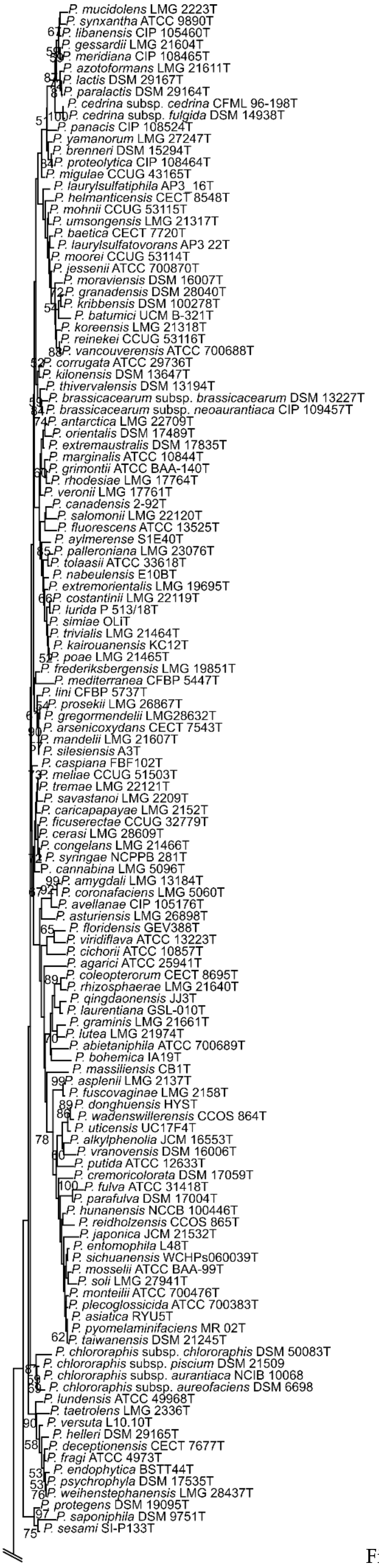

Figure S1A. Continued.

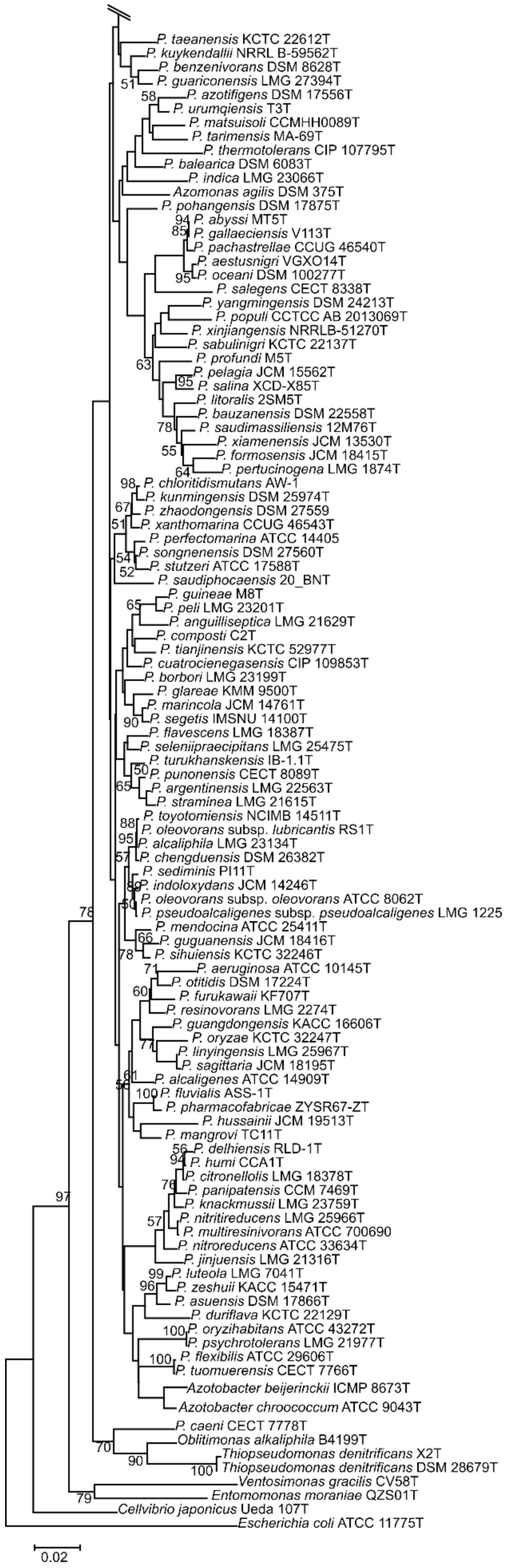

Figure S1. Phylogenetic tree based on the 16S rDNA sequences of the species and subspecies type strains under study. A) *P. fluorescens* lineage, B) *P. aeruginosa* and *P. pertucinogena* lineages. Bootstrap values higher than 50 % are indicated on the nodes.

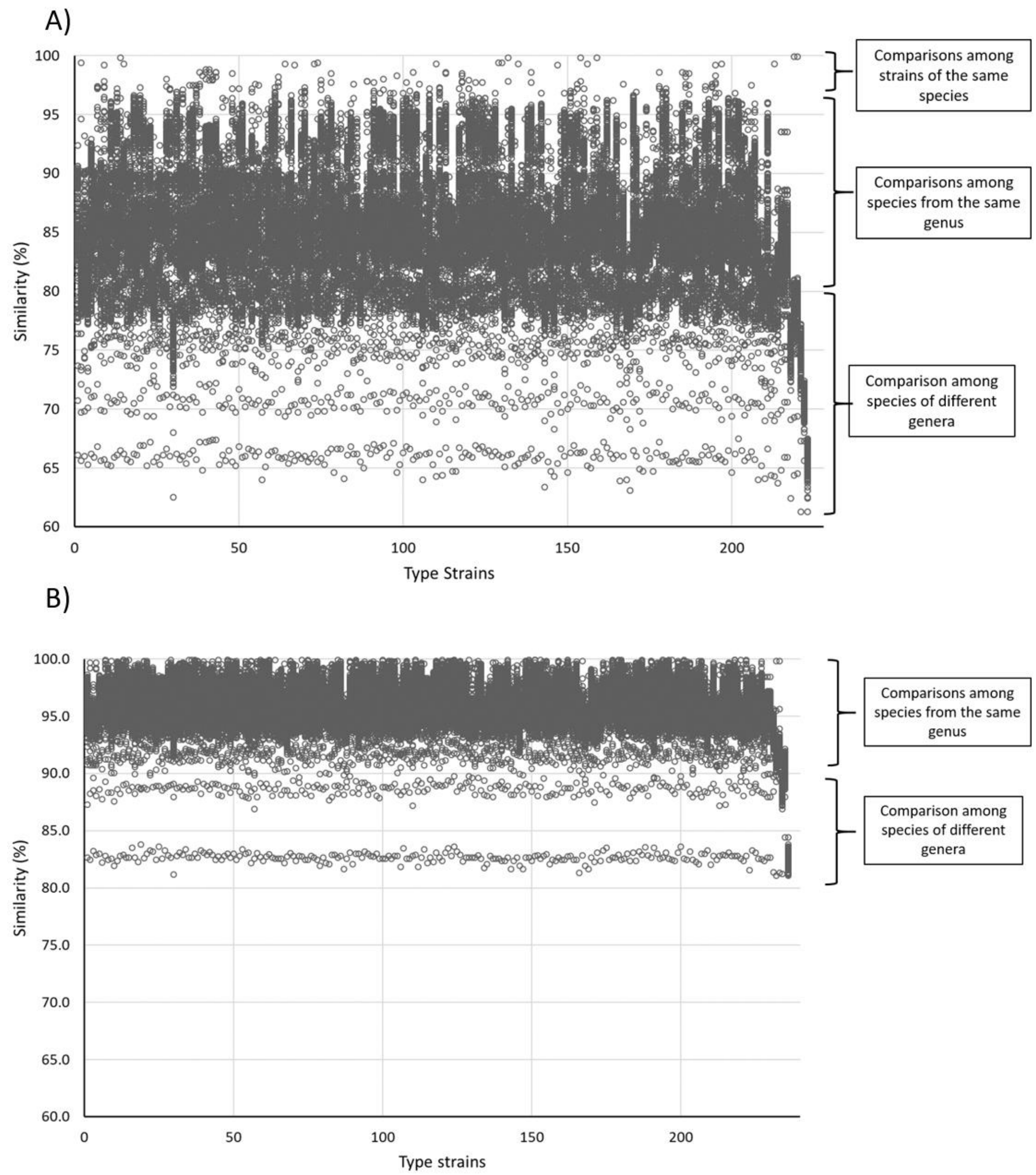

Figure S2. Pairwise similarities of the cocncatenated 4-genes partial sequences (A) and the 16S rDNA sequences (B) among the studied type strains.

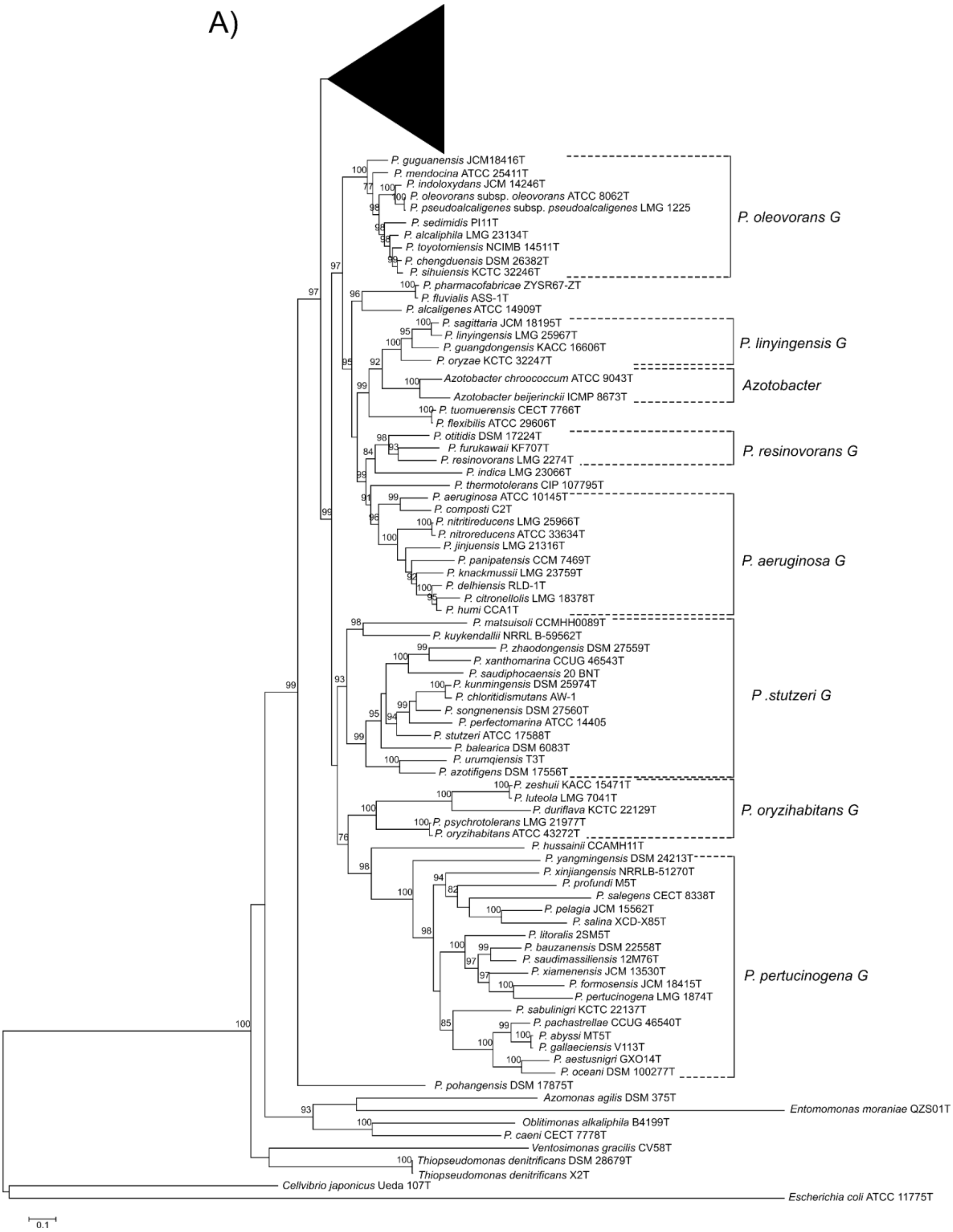

Figure S3A. Continued.

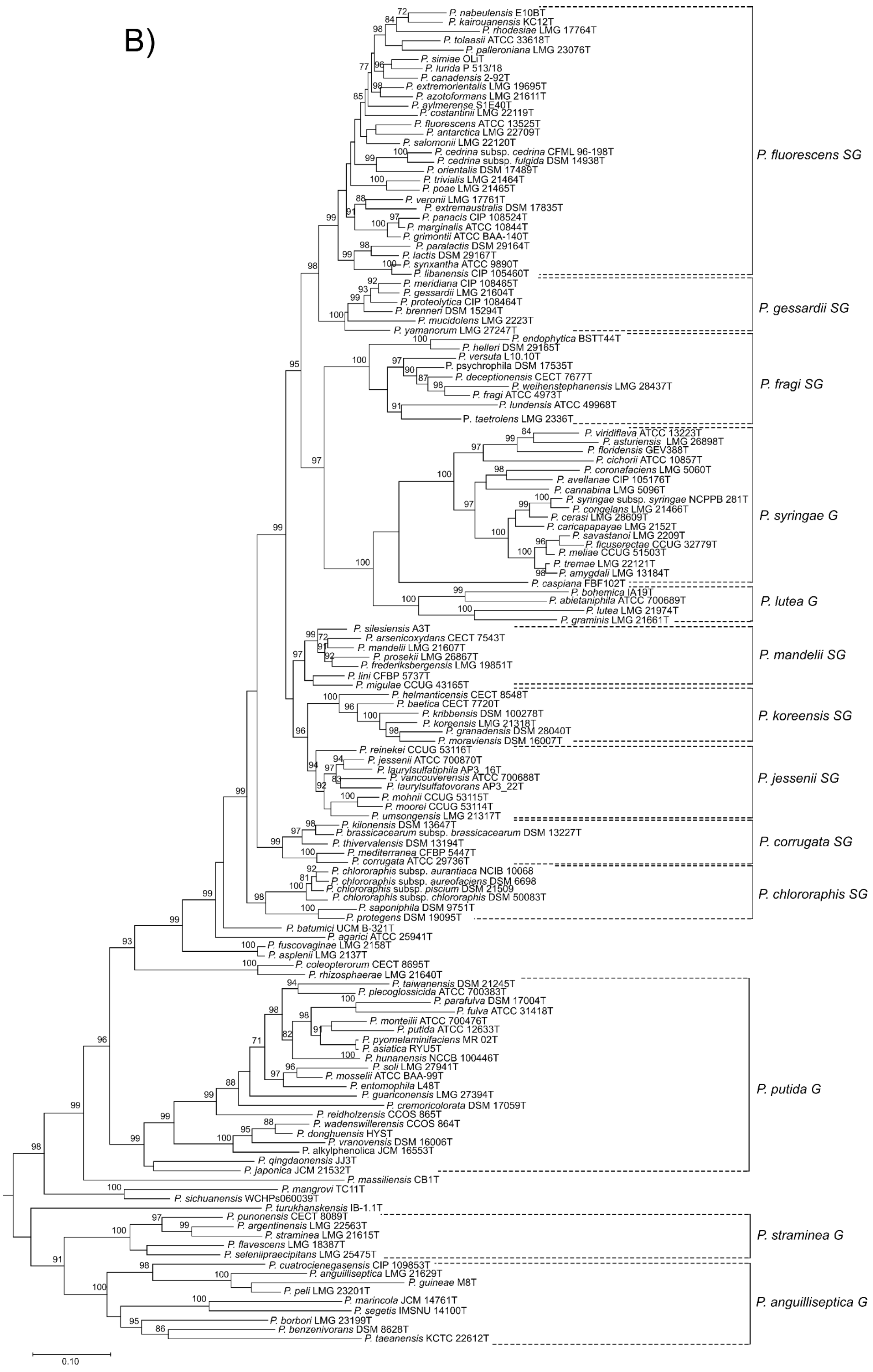

Figure S3. Maximum likelihood phylogenetic tree based on the 4-genes MLSA for the 241 species and subspecies type strains under study. A) *P. aeruginosa* and *P. pertucinogena* lineages. B) *P. fluorescens* lineage. Bootstrap values higher than 70% are indicated on the nodes. Bars indicate sequence divergence.

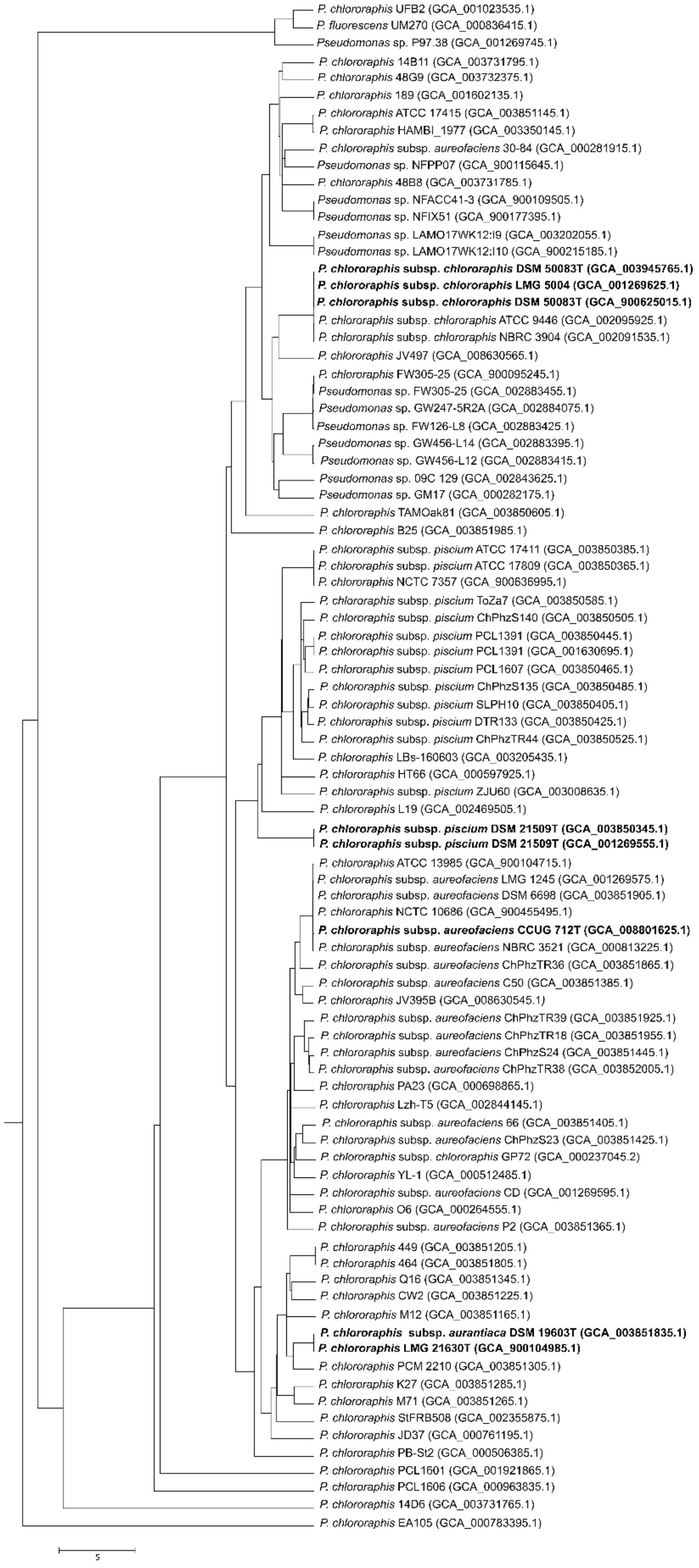

Figure S4. UPGMA dendrogram of the ANIb similarities among the *P. chlororaphis* strains studied.
